# Progress of the Surgery of the Vermiform Appendix

**Published:** 1903-09-19

**Authors:** 


### PROGRESS OF THE SURGERY OF THE VERMIFORM APPENDIX.

Appendicitis.—Barling[1] reports 143 cases operated upon in the acute stage with 25 deaths—that is, a mortality of 17·5 per cent. He considers that all these cases would have died if the disease had been left to run its course unless some unforeseen accident should have dispensed with the necessity for operation. In exceptional cases, with severe peritonitis, the temperature is normal or subnormal, and if we have the paradox of a falling temperature and an increasing pulse the former should be disregarded and the latter should be our guide to the patient's condition. If the appendix is not removed recurrence is quite uncommon, for in 25 of the cases in which it was not removed in only one was there recurrence. Lockwood[2] says no rules can be laid down for one of the most variable, complicated and treacherous diseases in the whole range of medicine and surgery. He prefers to remove the appendix whenever possible. A gangrenous or perforated appendix floating in thin stinking pus and surrounded by feeble and scanty adhesions, is just the one easily to be removed, and the case is just the one to end fatally with diffuse septic peritonitis. But an inflamed appendix surrounded by a little thick tenacious pus and hemmed in with dense tough adhesions, and perhaps hidden behind the right colon or in the pelvis, is calculated to baffle the most determined surgeon. But such cases are just the ones to recover, whether the removal of the appendix be attempted or not. The reward of so difficult an operation comes when we can assure our patient that he cannot have another attack of his dangerous malady. A peculiar case is mentioned by Damianos.[3] A patient had symptoms of appendicitis on the right side of the abdomen as usual, but on making the incision in the right iliac fossa no trace of cæcum or appendix could be found and pus welled up from the left side of the abdomen. The wound was therefore closed and a corresponding one made on the left. There was a normal descending colon and sigmoid flexure, immediately to the right of which were the cæcum and vermiform appendix, the latter surrounded with pus; the appendix was removed but death occurred from vomiting and collapse. Bloodgood[3] forms the following conclusions regarding the value of a blood-count in appendicitis:—1. In a suspected case before the fourth day, a rising leucocytosis indicates operation, *e.g.*, if the leucocytosis is 18,000 during the first 48 hours. If less than this, or if the leucocytosis is decreasing hour by hour, an operation is usually unnecessary. 2. After the fourth day a high leucocytosis indicates a localised abscess. If peritonitis is present there may be but slight leucocytosis, and this drops as the patient becomes more septic. If it is high there is a better chance of recovery. Hall[4] urges that in acute cases the fatal mistake is too frequently made of waiting for "positive evidence" of general peritonitis. He suggests the following as a reasonable line of action to adopt:—(1) When marked signs of peritonism do not ameliorate during the first six or eight hours after the onset, operate; (2) where acute local symptoms persist beyond the fourth day, operate; (3) where subacute or mild symptoms persist into the second week, operate; (4) when in doubt, operate. As to the frequency with which the more common individual organisms are found in the appendix, Eccles[5] says the following may be taken as a fairly accurate estimate, based as it is upon the combined results of the investigations of many authorities:—(1) Varieties of the bacillus coli communis, 94 per cent.; (2) bacillus pyocyaneus 0·5 per cent.; (3) bacillus prodigiosus 0·5 per cent.; (4) bacillus tuberculosis 2 per cent.; (5) staphylococci 1·5 per cent.; (6) streptococci 1·5 per cent. Barker[5] mentions the following conditions which have closely simulated appendicitis:—(1) Ruptured pyosalpinx; (2) ovarian cyst strangled by twisted pedicle; (3) twist and strangulation of omentum; (4) perforated gastric ulcer; (5) retro-cæcal hernia; (6) broken down caseating glands; (7) ilio-cæcal cancer with abscess; (8) hæmatoma of broad ligament; (9) reduction of hernia *en masse*; (10) intussusception. Lancien[6] records several cases of appendicitis in which bladder symptoms were important. Most usually there is retention, or micturition may be difficult, and accompanied by marked dysuria and abnormal frequency of micturition. In almost all the recorded observations of appendicitis accompanied by bladder trouble, the patients had acute appendical symptoms. In some of the cases symptoms of appendicitis were mixed, the bladder trouble being very marked. In such cases it is impossible to explain the vesical symptoms as due to a reflex irritation. It is more logical to believe that an inflammatory focus is the cause. Such cases sometimes end by an appendical abscess pointing into the bladder and setting up cystitis, pyuria or even pyelocystitis. A fistula may be permanently established, giving rise to communication between the bladder and an abscess cavity, or with the intestine itself directly or indirectly, according as this latter opens into the abscess cavity or directly into the bladder. In the case of stercoro-vesical fistula the urine contains pus, gas, and fæcal material. It is therefore important to note carefully any bladder symptoms arising in a patient who may possibly be the subject of appendicitis. Kelly[7] has discussed the question as to whether it is advisable to remove the apparently normal appendix during an operation performed for other reasons. He does not usually remove the normal appendix because:—1. The operation would slightly increase the risk of danger; shock might occur at any moment, and the additional five minutes consumed in the removal might have serious consequences. 2. There are no statistics to show that the existence of a normal appendix involves a definite risk per mille either of death or serious disease. 3. The appendix may have some function. In all right-sided pelvic operations he removes long free appendices, because an appendix which hangs free from the cæcum and is long enough to reach the field of an adjacent operation may become adherent and thus render the patient liable to an attack of appendicitis.

[1] Brit. Med. Jour., Jan. 10, 1903, p. 61. [2] Lancet, Dec. 13, 1902, p. 1608. [3] Med. Quarterly, May, 1903, p. 240. [4] Quarterly Med. Jour., Aug., 1902, p. 328. [5] Brit. Med. Jour., Feb. 28, 1903, p. 477. [6] Brit. Med. Jour., Jan. 10, 1903. Epitome, No. 18. [7] Lancet, Jan. 24, 1903, p. 254.

